# Supplementary figures and images for: Use of amplicon-based sequencing for testing fetal identity and monogenic traits with Single Circulating Trophoblast (SCT) as one form of cell-based NIPT
Source: PLoS One. 2021 Apr 15;16(4):e0249695. doi: 10.1371/journal.pone.0249695 (PMC8049273; doi:10.1371/journal.pone.0249695)

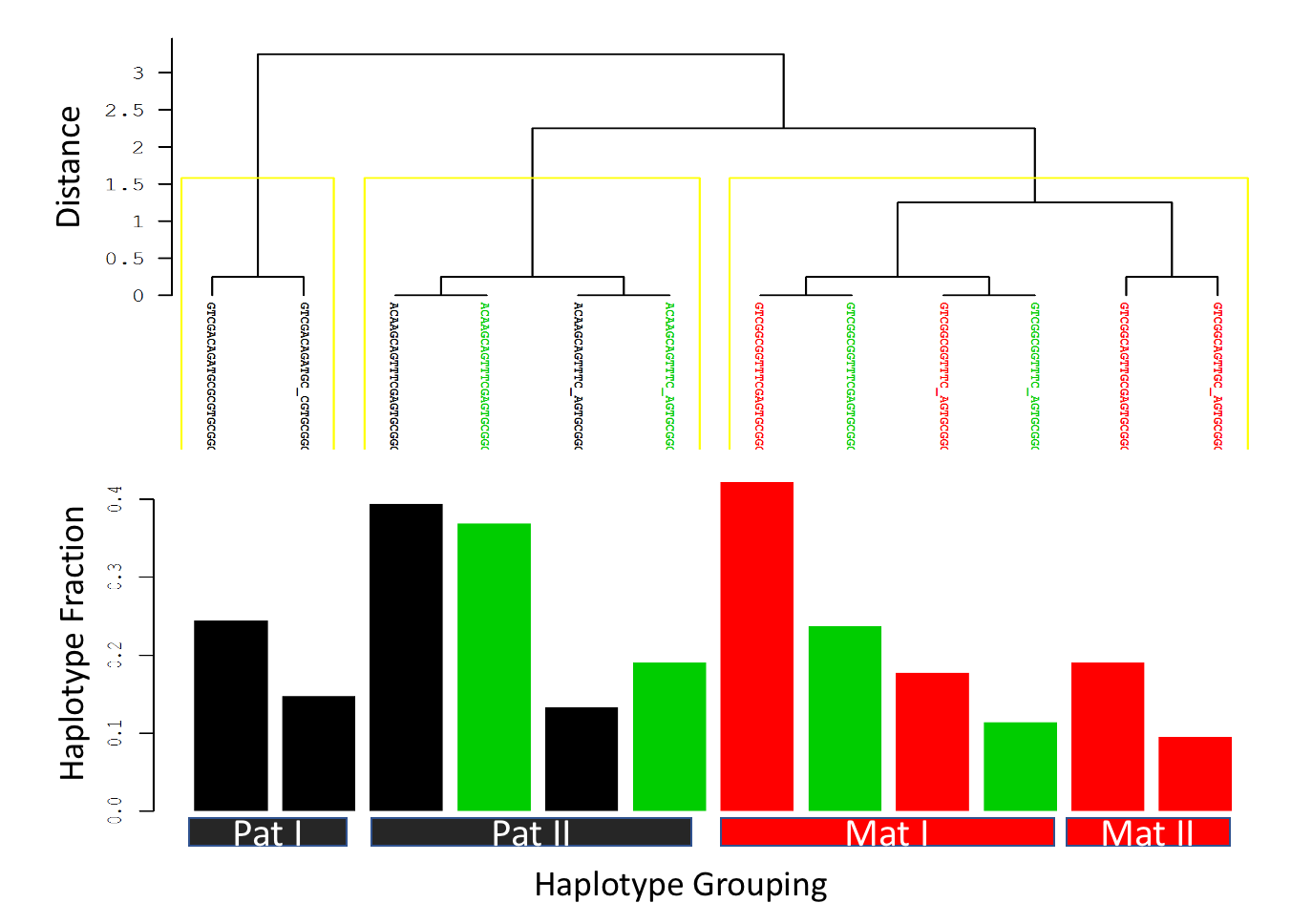

Supplement: S1 Fig — We use HLA-A amplicon haplotypes from samples present in Fig 6 to demonstrate how to identify the fetal cell. Haplotype groups of the mother (Red), fetal cell (Green), and father (Black). The Y-axis of bar graphs indicates the factions of total reads in each DNA types in different read groups. The tree cluster suggests the distance between read-groups according to Levenshtein distance calculation. (TIF) [file pone.0249695.s001.tif]

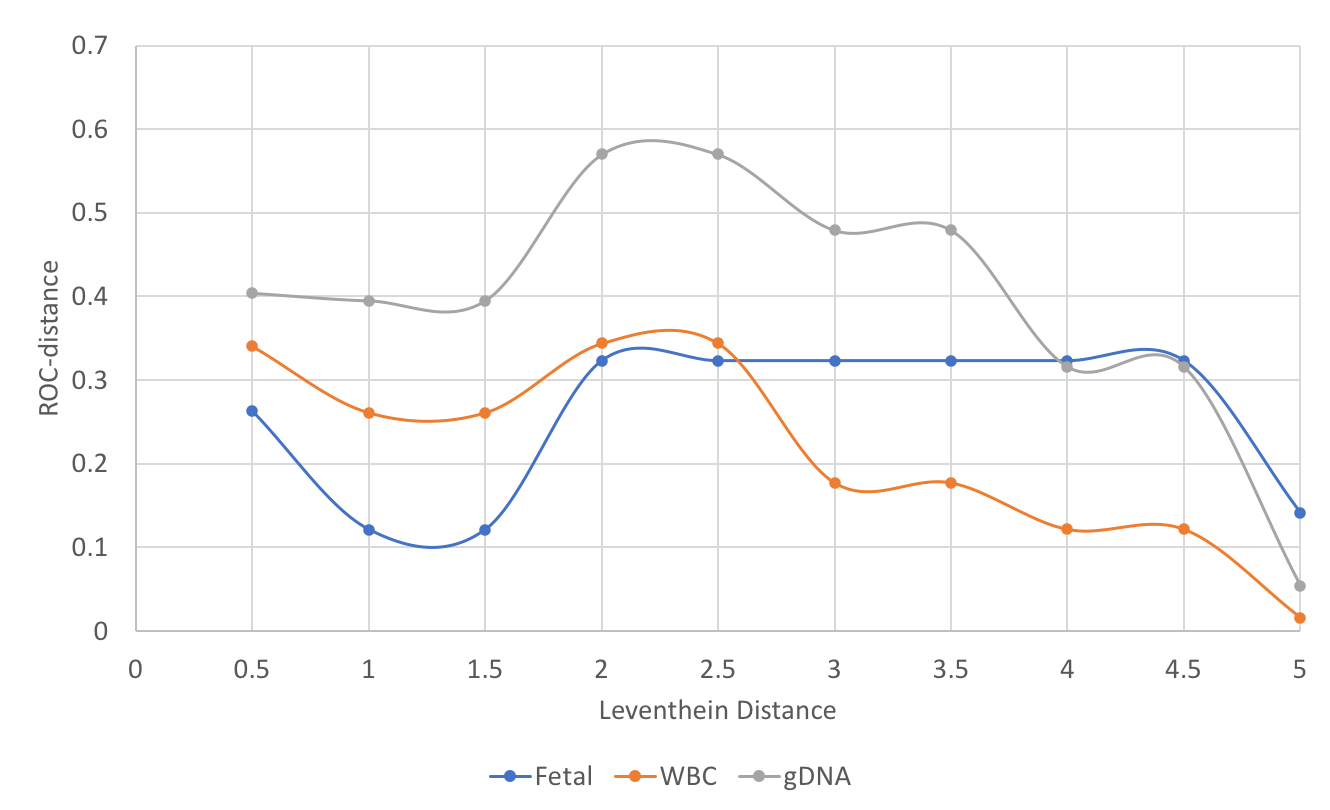

Supplement: S2 Fig — The Y-axis is the ROC-AUC distance to diagonal line from 0 to 1. The X-axis is the distance used for haplotype grouping. Three types of DNA were used for evaluation, Fetal (blue), WBC (Orange), and gDNA (grey). (TIF) [file pone.0249695.s002.tif]

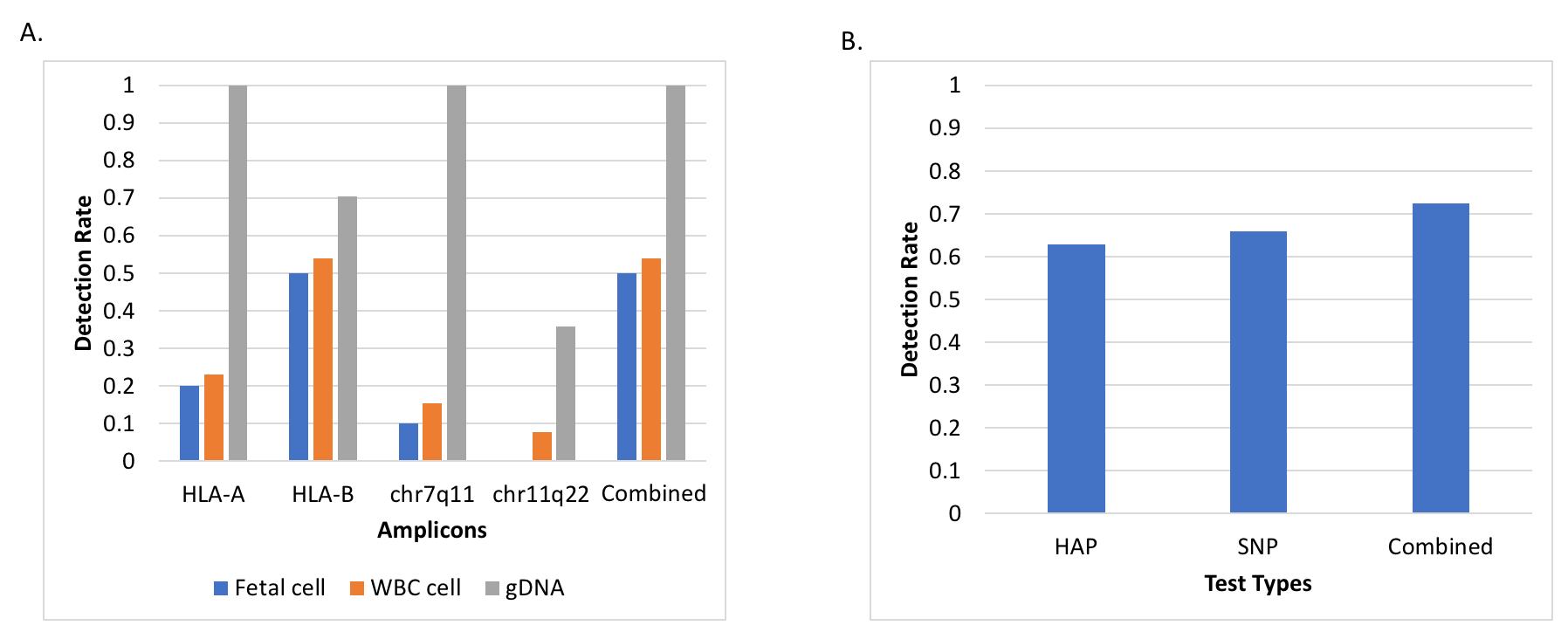

Supplement: S3 Fig — A. Performance of different haplotyping amplicons at detecting a non-maternal DNA. Y-axis is the detection rate, which estimates the fraction of the sample can be differentiated with a particular amplicon. The x-axis indicates which amplicon was tested. Fetal cells, WBC cells, and gDNA were tested accordingly from selected cases with both fetal cells and WBCs present. B. Improving detection rate by combining SNP typing and Haplotyping. WBCs from different cases (with or without fetal cells) were analyzed with individual and combined approaches. (TIF) [file pone.0249695.s003.tif]
