# Supplementary material for: Use of amplicon-based sequencing for testing fetal identity and monogenic traits with Single Circulating Trophoblast (SCT) as one form of cell-based NIPT
Source: PLoS One. 2021 Apr 15;16(4):e0249695. doi: 10.1371/journal.pone.0249695 (PMC8049273; doi:10.1371/journal.pone.0249695)
Supplement: Attachment — Submitted filename: rebuttal letter oct31.docx [file pone.0249695.s012.docx]

Dear Editor,

We would like to thank the reviewers for their specific and helpful comments. The manuscript has been improved according to the suggestions from the academic editor and reviewers.

For specific comments, please see our response below:

To Editor’s comments:

*1. Please ensure that your manuscript meets PLOS ONE's style requirements, including those for file naming.*

We changed the format of manuscript and naming of files to meet PLOS One Style.

*2. Please provide additional details regarding participant consent. In the ethics statement in the Methods and online submission information, please ensure that you have specified (1) whether consent was informed and (2) what type you obtained (for instance, written or verbal, and if verbal, how it was documented and witnessed). If the need for consent was waived by the ethics committee, please include this information.*

We provide additional details about consent in materials and methods. “Blood samples were collected from pregnant women from multiple centers under a protocol approved by the Baylor College of Medicine Institutional Review Boards utilizing written informed consent.” It is now indicated that this was written informed consent.

*3. We note that Figure(s) [1] in your submission contain copyrighted images. All PLOS content is published under the Creative Commons Attribution License (CC BY 4.0), which means that the manuscript, images, and Supporting Information files will be freely available online, and any third party is permitted to access, download, copy, distribute, and use these materials in any way, even commercially, with proper attribution.*

We redraw Figure 1 to address the conflict.

4. *Thank you for including the following competing interests statement; "The Houston authors are faculty and staff at Baylor College of Medicine (BCM), which is a partial owner of a for profit diagnostic company, Baylor Genetics (BG); Houston authors also are employee of or have advisory or lab director roles at BG. ALB is founder and CEO of Luna Genetics, Inc."*

*Please confirm that this does not alter your adherence to all PLOS ONE policies on sharing data and materials, by including the following statement: "This does not alter our adherence to PLOS ONE policies on sharing data and materials.” (as detailed online in our guide for authors http://journals.plos.org/plosone/s/competing-interests). If there are restrictions on sharing of data and/or materials, please state these. Please note that we cannot proceed with consideration of your article until this information has been declared.*

" The Houston authors are or were faculty and staff at Baylor College of Medicine(BCM), which is a partial owner of a for profit diagnostic company, Baylor Genetics (BG); Houston authors are also employees of or have advisory or lab director roles at BG. ALB is founder and CEO of Luna Genetics, Inc. This does not alter our adherence to PLOS ONE policies on sharing data and materials.”

To Reviewer #1’s comments:

The paper “Use of amplicon-based sequencing for testing fetal identity and monogenic traits with single circulating trophoblast (SCT) prenatal diagnosis” by Zhuo et al. is well written and clearly describes a promising procedure to distinguish fetal from maternal cells. However, I have a few remarks that require clarification.

*Major point*

*- There is no discussion on the presence of fetal cells still present from previous pregnancies. Fetal cells have been detected decades after a pregnancy. Can the method described also distinguish if a fetal cell has a different haplotype than other fetal cells? If so, this is a strength of the methods described. If not, this would be a limitation.*

*- In addition, what would be the effect of twin pregnancies?*

*- Extra discussion is needed on the effects of the analysis on downstream analyses. Does the procedure cause lower quality results in downstream analysis of selected cells, since extra WGA amplification is needed to perform this test.*

The following is added to the Introduction. This genotyping uses a small aliquot of the WGA product and does not interfere with downstream analysis.

To the discussion. Although there is evidence that some cells from previous pregnancies can persist for decades, especialy CD34+ cells,{PMID: 8570620} there is no evidence that trophoblasts can persist from previous pregnancies, and we expect based on the biology of these cells including apoptosis, that they are unlikely to persist. A future method that provides deeper genotyping of individual cells could distinguish cells from previous pregnancies and cells from same sex nonidentical twins, but the method described here would not be sufficient to accomplish such distinction reliably.

*Minor points,*

*- Line 999: Settings used for BWA-MEM. Does ‘conventional BWA-MEM’ mean all default settings?*

Since it is amplicon seq and the data size relatively small, we can do the mapping on a regular laptop rather than a server, so we adjusted the BWA-MEM setting for small memory and minimum seed length (see the supplement script S3 for exact setting).

*- Table 1 is rather a list of amplicons containing SNP regions than a list of SNPs.*

The title of the table has been changed to List of Amplicons

*- In table 1: the start or stop of the HLA-A amplicon seems to be incorrects (Start>stop and not matching amplicon size)*

Here we used the 1-base format rather than 0-base (1-base format include the first and last base of the amplicon so that subtraction of one number from the other gives a number one less than the product size), so user can be easier to access the location in UCSC genome browser (1-base also). I add a footnote to the table to clarify the format.

*- Lines 321 and 322, perhaps add the Protein accession numbers to the variants*

We added ClinGen accession number to the variant in figure legend.

*- One method to infer haplotypes in cfDNA was not yet mentioned.* [*https://pubmed.ncbi.nlm.nih.gov/28844486/*](https://pubmed.ncbi.nlm.nih.gov/28844486/)

We add this article to the reference:16. Vermeulen C, Geeven G, de Wit E, Verstegen MJAM, Jansen RPM, van Kranenburg M, et al. Sensitive Monogenic Noninvasive Prenatal Diagnosis by Targeted Haplotyping. Am J Hum Genet. 2017;101(3):326-39. Epub 2017/08/24. doi: 10.1016/j.ajhg.2017.07.012.

*- Spelling mistake in fig 4: mother  mother and (informatic  informative?)*

We fixed this typo.

*- The github code would benefit from some polishing, adding comments and removing commented out codes.*

We have made revisions to address this.

To Reviewer #2’s comments:

*In this paper, the authors study the application of WGA followed by SNP and / or haplotype analysis in multiple amplicons to determine the fetal or maternal origin of presumably fetal cells, retrieved from the maternal blood. They do so by using different amplicons, i.e. amplicons from regions such as the HLA regions, with multiple SNPs per amplicon for haplotyping, and Human Identification SNP test amplicons, with one SNP per amplicon. They also studied the possibility of using these methods for genotyping for monogenic traits. Below my comments and questions:*

*1. My main comment is that, even though the authors do mention the drawbacks of their method, the paper is quite optimistic. The theory behind the methods they describe is solid, but the actual data are still quite preliminary. In their abstract the authors say that the method allowed reliable differentiation between fetal and maternal cells, in the introduction in line 75 they mention “in most cases”, but this was only in cases with sufficient information, which is certainly not 100%. Not all data is presented in a clear way, such as numbers of cells that are informative, but in how many cases? See my comments below. The methods that are described certainly have potential for this application, but need to be improved and the authors do acknowledge this in the discussion. In the discussion, the authors do mention multiple options for improvements, but how feasible are these? If feasible, why didn’t the authors already implement these, or at least show some data of improved versions, as proof-of-principle. The authors show data on fetal genotyping for monogenic traits, but in the discussion, they mention themselves that a low failure rate is to be expected due to failure to isolate fetal cells and/or to ADO.*

The following sentences were added to the discussion, acknowledging the points made by the reviewer.

“The data demonstrate the principle that detailed genotyping of individual cells can distinguish fetal from maternal cells, but the data are somewhat limited and preliminary, and new methods to more extensively compare the genotype of individual cells to the genotype of the mother are needed.”

The text has been modified as follows to address allele drop out and failure rate.

“Single cell analysis of circulating trophoblasts can be used to determine the genotype of fetuses at risk for monogenic disorders, although allele dropout must be ruled out by analysis of multiple cells or it can be addressed using karyomapping as has been used in single cell preimplantation testing.{PMID: 19858130} Hopefully improved methods for recovering fetal cells from mother’s blood will reduce any failure due to lack of cells, but use of cell-free NIPT in combination with haplotype analysis or digital PCR, amniocentesis, and CVS would be three alternative strategies for difficult cases. “

*2. Throughout the paper, the authors use the term cell-based NIPT, whereas in the title they use the term single circulating trophoblast prenatal diagnosis. I would suggest to use the same terminology throughout the paper, and would suggest to also use the term cell-based NIPT in the title.*

Single circulating trophoblast testing is one form of cell based NIPT. They do not mean the same thing. We have modified the title to clarify this. Fetal nucleated RBC-based NIPT would be another form of cell based NIPT.

*3. Line 40: analysis cell free = analysis of cell-free*

Fixed

*4. In the introduction, the authors mention some drawbacks of cf-NIPT. One major drawback, of course, is the fact that cf-NIPT studies cd-DNA from the placenta and not from the fetus itself. This major drawback is not solved by the cell-based technique the authors use, as trophoblast cells are used for this method. The authors should comment on this. Furthermore, even though it is outside the scope of this paper, in the introduction the authors should also (briefly) comment on drawbacks of the cell-based NIPT, such as costs as compared to cf-NIPT and high-throughput possibilities.*

There are many complexities around many different forms of testing and mosaicism. The following sentence has been added to the introduction. “Cell-free NIPT, trophoblast-based NIPT, and CVS all can detect placental mosaicism, while amniocentesis, fetal nucleated red blood cell (fnRBC)-based NIPT when feasible, and fetal blood sampling can help to clarify whether mosaicism involves the fetus or is confined to the placenta.”

The following was also added. “Limitations of cost and throughput would need to be overcome for cell-based NIPT to be a routine alternative. At present to our knowledge, no laboratory offers cell-based NIPT as a clinical test.”

*5. With SNP typing, DNA from 156 blood samples were compared, with data from 518 cells: 357 cells were informative. Does being informative mean that the cells were fetal, or were there also maternal cells identified? If all fetal, were these cells originally also from 68.9% of the 156 samples, or were cells from some samples overrepresented and others underrepresented? In other words, could informative (fetal?) cells be retrieved from 68.9% of the 156 samples? (This refers to the comment that not all data support the conclusions, questions 1 and 3 of the review).*

The following sentence has been added as noted above in response to similar comments earlier. “The data demonstrate the principle that detailed genotyping of individual cells can distinguish fetal from maternal cells, but the data are somewhat limited, and new methods to more extensively compare the genotype of individual cells to the genotype of the mother are needed.”

The following has been added. “The results for cases can be divided into four groups as shown in Table 2. First, there were 7/154 (4.5%) cases where none the cells passing WGA could be proven to be fetal; Second, there were 42/154 (27.3%) cases with only one cell scored as fetal; and third, there were 103/154 (66.9%) with two or more cells scored as fetal as defined in methods. At the single cell level for cells that pass WGA, cells can be scored in only two ways: 1) uninformative meaning that no or inadequate evidence of fetal status or 2) adequate evidence for fetal status. Some of the “uninformative” cells were certainly maternal, and we cannot distinguish an uninformative fetal cell from a maternal cell. Deeper genotyping would make healthy uninformative fetal cells informative, but some fetal cells are known to be apoptotic with degraded DNA and may or may not pass WGA.” Case number reduce from original 156 to 154, due to 2 cases have poor quality maternal gDNA, which hinder the analysis.

*6. For haplotyping, how many cells from how many samples were tested? Again 156 samples? Also here, were the informative cells distributed evenly over the samples. In how many samples could fetal cells be identified based on haplotyping only? Is 50% of all cells the same as 50% of the cases? (Again, see questions 1 and 3 of the review)*

Insertion of new Table 2 addresses this.

Table 2. Tabulation of cases according to outcome of genotyping.

| Condition | 2 SNP + 10% | 2 SNP + 8% | 2 SNP + 6% | 2 SNP |
| --- | --- | --- | --- | --- |
| None of the available cells with adequate WGA were proven fetal. | 13 | 8 | 7 | 7 |
| Only one fetal cell identified. | 46 | 44 | 42 | 42 |
| Two or more fetal cells identified. | 93 | 100 | 103 | 103 |
| Total cases | 154 | | | |

*7. The authors state they tested the haplotyping method with matched gDNA, WBC and fetal cells. In Line 278, they only mention WBC, not the fetal cells. What is the differentiating rate for the fetal cells? Please mention this in the text, not only in a supplementary figure, as this is the main subject of this paper. Moreover, according to Figure S3A, this is about 50% for both WBC and fetal cells, and this percentage is determined by one amplicon (HLA-B).*

Both the text and figure S2 and S3A mentioned fetal cells with WBC (Maternal cell). Since the Haplotyping only offer relative smaller contribution to the differentiation than SNP typing, we keep them in the supplement.

The Figure S3B were used to estimate the power of a different genetic origin cell, WBC(single Maternal cell), from unmatched gDNA. Because the WBCs is picked intentionally and went through all the processing steps as potential fetal cells, which offer two benefits as control. First, their origin is very certain maternal origin. Second, they usually preserve a better shape, in contrast fetal cells occasionally were in the process of apoptosis or aggregated together. Comparing WBC with unmatched gDNA allow us to compare and estimate the limit of our method when encounter an intact cell.

*8. Figure S3A shows fetal cells with a detection rate of about 50%. In Figure S3B for HAP this seems to be more than 60%. What is the difference?*

First, S3B for HAP is comparing the detection rate of WBC (~62%); while in S3A, WBCs combined is about 55%, they are about 7% difference. Second, S3A use only case with both Fetal cell and WBCs. S3B include cases with or without Fetal cells. Because they use different groups of cases for calculation, they cannot be compared directly.

We edit the text as following:” when four amplicons were combined, we can still distinguish about 50% of all the cells (from selected cases with both fetal cells and WBCs) (S3 Fig A). With combined power of both SNP typing and haplotyping, we can increase the solving rate of differentiating a WBC from around 60% to more than 70% (from cases independent of fetal cell existence) (S3 Fig B).”

*9. Lines 302-304: the authors state that 5 cells were genotyped: two were heterozygous and two had ADO. What happened to the fifth one?*

The fifth cell does not carry mutation on that position. It is a possibility that the mutant allele drop out in this cell.

*10. In line 288, the authors state that in the cultured lymphoblasts, the ADO rate was 15% and 8%. When using true fetal cells from the three families, the ADO is very much higher, about 50%, as in each family some cells show ADO. How do the authors explain this difference?*

Sentence added. “The higher rate of allele drop out in fetal cells is presumably caused by some combination of DNA degradation caused by time in the maternal circulation including apoptosis and by cell isolation including fixation and permeabilization steps.”

*11. From two of the three families more cells were retrieved than were used for genotyping. Why did the authors not genotype all available cells? Could not all cells be identified as true fetal? If so, that the identification of true fetal cells is lower than 60-70%. How were these cells identified as true fetal? If the other cells were maternal in origin, the chance of picking up a maternal cell is much higher than 10%, as stated in the introduction in line 65.*

We do a quality check after WGA and determine if the cell should move on the next step. For cells pass the QC, we do the genotyping for most of them. Some cells either suffer bad quality (very low read number in sequencing) or have dropout reads in the position (such as cell dicussed in question #9).

To Reviewer #3’s comments:

*This paper describes the use of SNPs genotyping by sequencing in order to identifiy circulating cells of fetal origin in the context of NIPT applications.*

*Although the subject matter and the approach are interesting, there are significant issues with the way some of the results are presented and especially with the conclusions reached by the authors.*

*Because of the major limitations of the proposed methods, highlighted by several results (e.g. the combined performance of SNPs genotyping and haplotyping that gives only a 70% rate of success for identifying fetal cells from WBC (line 278, fig S3 B), a high rate of allele dropout and amplification artefacts contributing to false-negatives and false positives (line 335-341)), a clear and detailed presentation of false positive/negative rates should be provided.*

Based on this comment and similar comments above, we made a new table2. Significant modifications have been introduced into the Results and Discussion as below.

“The results for cases can be divided into four groups as shown in Table 2. First, there were 7/154 (4.5%) cases where none the cells passing WGA could be proven to be fetal; Second, there were 42/154 (27.3%) cases with only one cell scored as fetal; and third, there were 103/154 (66.9%) with two or more cells scored as fetal as defined in methods. At the single cell level for cells that pass WGA, cells can be scored in only two ways: 1) uninformative meaning that no or inadequate evidence of fetal status or 2) adequate evidence for fetal status. Some of the “uninformative” cells were certainly maternal, and we cannot distinguish an uninformative fetal cell from a maternal cell. Deeper genotyping would make healthy uninformative fetal cells informative, but some fetal cells are known to be apoptotic with degraded DNA and may or may not pass WGA. False positives and false negatives at a single cell level are discussed below. This method is a work in progress, and we consider one fetal cell as partial success and two or more fetal cells as success from a clinical perspective. Improved cell recovery and improved genotyping would be needed to achieve an optimal test. The relative roles of failure to isolate and amplify cells, uninformative genotyping, and suboptimal numbers of fetal cells can be calculated from Table 2.”

Table 2. Tabulation of cases according to outcome of genotyping.

| Condition | 2 SNP + 10% | 2 SNP + 8% | 2 SNP + 6% | 2 SNP |
| --- | --- | --- | --- | --- |
| None of the available cells with adequate WGA were proven fetal. | 13 | 8 | 7 | 7 |
| Only one fetal cell identified. | 46 | 44 | 42 | 42 |
| Two or more fetal cells identified. | 93 | 100 | 103 | 103 |
| Total cases | 154 | | | |

*Unfortunately, these rates are not comprehensively and clearly presented in the paper. Table 2 is not clearly explained. For example, I'm puzzled by the apparently negative correlation between true positives (TPV) and % of SNP differences (diff SNP%) in the "Fetal cell" column, shouldn't it be the opposite ? More info about the table headers, interpretation, etc. should be given. One of the main focus of the paper should be to present, analyse and discuss these rates in details.*

We agree that the rates were no presented clearly, and we have constructed a different presentation with a new table 3, which we believe is much improved.

The following is inserted in the results.

“If one assumes that the NGS data are 100% reliable for sex based on X and Y data, which we believe is the case, the sensitivity and specificity of the genotyping can be estimated from normal male singleton pregnancies as shown in Table 3. Definition of other contingencies are included in the same table. Identifying a fetal cell as fetal by genotyping and confirming that it is male based on NGS is a true positive. The results vary depending on the cutoff for scoring a SNP allele as present. If we accept two different 2 SNP + 6% cutoff, based on normal male singleton pregnancies the true positive rate was 82.1%, true negative 6.6%, false positive 3.3%, and false negative 7.9%. This is equal to a sensitivity of 91.2% and a precision of 96.1%. These results would not be sufficient for clinical diagnosis, but they demonstrate the general validity of the approach and suggest that deeper genotyping of single cell could be completely reliable.”

Table 3. Sensitivity and specificity for genotyping

| **Normal male singleton** | | | | |
| --- | --- | --- | --- | --- |
|  | **True positive** | **True negative** | **False positive** | **False negative** |
|  | Cells scored as fetal by genotype and male by NGS | Cells scored as not fetal by genotype and female by NGS | Cells scored as fetal by genotype but female (maternal) by NGS | Cells scored as not fetal by genotype but male by NGS |
| 2 SNP | 126 | 10 | 5 | 10 |
| 2 SNP + 6% | 124 | 10 | 5 | 12 |
| 2 SNP + 8% | 117 | 10 | 5 | 19 |
| 2 SNP + 10% | 107 | 10 | 5 | 29 |

*This relatively poor performance of the genotyping approach to confirm fetal origin seems to be caused at least in part by the low amount/quality of starting DNA material since the detection rate for genomic DNA is much higher (Fig S3 A). This impact of starting material is also evident from the much lower number of scorable SNPs from WGA products as compared to genomic DNA shown on fig. 2.*

The reviewer is correct. The poor performance on genotyping is caused in part by low amount/quality of DNA.

The following sentence has been added “The higher rate of allele drop out in fetal cells is presumably caused by some combination of DNA degradation caused by time in the maternal circulation including apoptosis and by cell isolation including fixation and permeabilization steps.”

*These results thus point to serious technical challenges associated with WGA from single cells. Moreover, as the author acknowledge at line 335-341, both a high rate of allele dropout and amplification artefacts contribute to false-negatives and false positives.*

One can address false positive and false negatives theoretically in genotyping a single cell. False positives where a sequencing error introduces the alternative of two alleles at a position would be very rare where alternate allele appears by chance. Single base sequencing errors are moderately common but do not create a false positive. False negative could occur due to allele drop out which is higher in cells recovered from mother’s blood than for lymphoblasts. Clearly ADO is a very common occurrence.

Limitations are failure to isolate fetal cells, failure of WGA (e.g., due to apoptosis), allele drop out, and insufficient depth of genotyping. We hypothesize that all of these can potentially be overcome by recovering more cells and by deeper genotyping of each cell (i.e. genotyping thousands of SNPs).

*The allele dropout problem mentionned above was also seriously affecting the genotyping for monogenic diseases, with rates reaching 8-15% (line 288). Such rate is clearly incompatible with any diagnostic application.*

ADO is a problem for diagnosing monogenic disorders using cell-based NIPT. If a nucleotide position is heterozygous in even one cell, but preferably in multiple cells, the genotype at that position can be scored with confidence. If the genotype shows only the normal (N) allele at a position, interpretation can conclude that the genotype is NN or NM but not MM (affected). If the genotype shows only the mutant (M) at a position, interpretation can conclude that the genotype is MM of NM but not NN. This circumstance occurs for common variants causing phenotypes as for sickle cell anemia or cystic fibrosis. If one allele is found in some cells and the opposite allele is found in other cells in a singleton pregnancy, we would argue that finding two cells with only the N allele and two cells with only the M allele would allow the conclusion that the genotype is heterozygous at that position. For compound heterozygous genotypes, the circumstances are somewhat different. Observing both alleles in multiple cells is straight forward for interpretation that the fetus carries the mutant allele at this position. Observing only the M allele would also allow the conclusion that the fetus carries the mutant allele at this position Observing only the N allele in even many cells is challenging, since the fetus could carry the mutation with allele dropout in all five cells. This could occur especially if a SNP impairs the function of a primer. This concern can be reduced by demonstrating that the primers used amplify all parental alleles. The uncertainty of a genotype position could be addressed by cell-free NIPT for a paternal mutation, but a method such as haplotyping, karyomapping, digital PCR, or amniocentesis or CVS could be used for maternal mutations.

*From these results, the realistic take home message is clearly one of skepticism towards the feasibility of using this kind of approach clinically in the short to mid term. However, although the authors acknowledge some of these limitations in the discussion, they nevertheless make statements that I consider misleading with respect to what the data show. For instance, "This single cell genotyping assay can provide an essential step for confirming the fetal origin of cells obtained from cell-based NIPT workflows. Through genotyping, we can reject cells that are of indeterminate or maternal origin and provide metrics for WGA DNA quality using multiple amplicons." (line 328-331). "...this assay is rapid and reliable..." (line 335). "Use of this method for genotyping fetuses at risk for specific monogenic mutations is feasible." (line 352-353).*

We agree with skepticism about using the current method clinically, but it does demonstrate that a deeper genotyping method that detects thousands of SNPs in single cells could be used clinically.

*More details should also be provided about the exact number of amplicons used rather than vague statements such as: “We sequenced approximately 90 highly polymorphic SNPs within about 40 amplicons.” (line 72). Likewise, in the methods section, three groups of amplicons are mentioned but only for the second group, a number is provided (37), while a reference is offered for the first group (Debeljak et al.), no number is given concerning the third group. Table 1 listing the amplicon used contains 49 entries.*

The total number of amplicons are case specific. First 41 amplicons are regular for identifying the origin of cell. Then additional amplicons are added if the parents have monogenic disorder, so the total number is case by case.

*Overall, the way the methods and results are presented lacks clarity and details that complicates the evaluation of the technique.*

*I thus consider that in its current form, this paper suffers from 1) a lack of clarity and a lack of emphasis on false positive/negative rates and other performance statistics, and 2) needs a more sober and realistic interpretation of the results, less focused on optimistic hopes of better future performances and more concerned about current challenges.*

We think that the revisions above address these weaknesses.

We hope that our revision in its current form is suitable for publication in PLOS ONE.

Sincerely yours,

On behalf of the authors

Xinming Zhuo, Ph.D. and Arthur Beaudet, M.D.
