# Supplementary material for: Use of amplicon-based sequencing for testing fetal identity and monogenic traits with Single Circulating Trophoblast (SCT) as one form of cell-based NIPT
Source: PLoS One. 2021 Apr 15;16(4):e0249695. doi: 10.1371/journal.pone.0249695 (PMC8049273; doi:10.1371/journal.pone.0249695)
Supplement: Attachment — Submitted filename: reply_to_comment.22Jan2021.docx [file pone.0249695.s013.docx]

Dear Editor,

We would like to thank the reviewers for their specific and helpful comments. The manuscript has been improved according to the suggestions from the reviewers.

For specific comments, please see our response below:

Reviewer #2: The authors have done a significant amount of work from which the paper certainly benefits.
Previously, I commented on the difference between cells and cases. Even though the data in the paper are presented in a much better way now, I still have some questions:
1. In the rebuttal the authors mention the number of cases is now 154 instead of 156. In the text, they use the number of 154, while in table 2 the numbers do not add up to 154 but to 152. Please explain or correct.

The following sentence is added to the first paragraph of methods describing the samples.

“There were 154 usable blood samples from 152 pregnancies/cases; two women had two samples collected during one pregnancy.”

To avoid confusion, we also revised the number accordingly in the text.

2. In Table 2, numbers of cases are presented, which in my opinion is indeed the most informative way of (at least) presenting the data. In lines 213-218 the authors again switch to information on number of cells instead of cases. This apparently is not the same as cases, as when calculating % from the data in Table 2, different % are obtained than mentioned in the text. Please use the same way of presenting data in both text and Table.

The paragraph starting at line 213 is substantially rewritten as follows:

In Table 2, we examined what percent of cases had one or more or two of more cells scored as fetal. Individual cells were scored as fetal if two or more SNPs had at least 10% reads for an allele that was not present in the mother. Cases were then subdivided into those where the informative SNPs indicating fetal status were at least 10%, 8%, or 6%, of the scorable SNPs. There were no cases where the informative SNPs were less than 6% of the scorable SNPs. There were 60 cells with one informative SNP suggesting that requiring two SNPs may undercount fetal cells. It is important to distinguish the percent of cases (82.4%) that had two or more fetal cells (103/152) from the percent of cells (78.7%) that had two or more fetal SNPs indicating fetal status (408/518).

3. In Table 2 the last column seems to be the same as the “2 SNP + 6%” column as far as data is concerned. What does this last column show? According to Table 3 it should be “2 SNP + 0%”, but what does that mean? The numbers in the column in Table 2 are the same as in the “2 SNP + 6%” column. Is this correct?

The last column of Table 2 has been deleted as not contributing useful information.

4. Table 2 would benefit from mentioning also the percentages, for better comparison with the text.

We edited the table as suggested.

5. Lines 226-230 and Table 3: this is again based on cells. Please also provide information of cases.

This Table presents the reliability of scoring a cell as fetal, and it is essential to present the data as cells. There are multiple cells per case, and it is not the case that is a false positive or false negative. No change is made to the text.

6. Lines 337-342: As mentioned in my previous comment, the data n the numbers of cells do not add up to the numbers of cells that are genotyped. Please add information on all the cells, for both the DHCR7 and the sickle cell family.

For the sickle cell case, we made an error. Nine fetal cells were recovered and four were genotyped. The fifth cell was a maternal WBC intentionally picked as a control. The text is corrected. Information for all of the cells genotyped is now presented for the DHCR7 and sickle cell families.

7. Line 337: “…but the data are somewhat limited…”. Please remove the word “somewhat”, as this single word raises a lot of questions and removing the word does not change the message of the sentence.

The word was removed.

8. Lines 403-405: the authors suggest as alternatives to overcome the problem with their method to use their NIPT in combination with amniocenteses and CVS. This seems a strange alternative, as cell-based NIPT is being validated and optimized as replacement for invasive methods. What would be the benefit of cell-based NIPT if it should be used in combination with an invasive procedure anyway?

The intent was to mention amniocentesis and CVS as alternatives if cell-based NIPT fails. The text is changed to indicate this.

Minor comments:
9. Abstract line 26: “This method allowed reliable differentiation of fetal and maternal cells.” This is still an optimistic, and possibily misleading, sentence. The sentence can be removed without any damage to the abstract.

Removed.

10. Throughout the paper, the term “mutation” is used. There is an international agreement to use the word “variant” or “pathogenic variant” instead (see Richards et al. 2015). Please use this throughout the paper.

There were 43 occurrences of the word mutation and 8 occurrences of the word mutant. Dozens of changes were made where appropriate.

11. Line 199: “…where none the cells…” = “…where none of the cells…”

Changed

12. Line 202: “…meaning that no…” = “…meaning no….”

changed

13. Line 214: “…used a more conservative….” = “…used a more conservative approach of…”

The entire paragraph was rewritten.

14. Line 226: “…two different 2 SNP…” = “two different SNPs…”

Changed

15. Line 241: “…per sample…” = “…per cell…”

changed

16. Line 351: “…the sickle mutation…” = “…the sickle cell anemia variant…”

Changed

17. Line 377: spelling error in pregnancies and piology (=etiology??)

Changed to biology

Reviewer #3: Although the authors have attempted to address all the comments raised in the previous review, a few issues persist.

Notably, in my opinion, the discussion still contains overly optimistic statements that do not adequately portrait what the current data suggest. For instance, at line 381, I would replace: "...this assay is rapid and reliable…" by "...this assay is promising…".
at line 393, replace: "...monogenic mutations is feasible." by "...monogenic mutations is potentially feasible.". At line 410, replace: "An improved version of this assay may significantly reduce…" by "A future improved version may hopefully reduce…".

Changed as suggested.

The paper would also greatly benefit from clarifying the terminology used when referring to "informative" cells and SNPs throughout. It seems like the authors refer to SNPs with more than one allele present in the NGS data from a sample as an informative SNP, whether or not there's a difference between the mother and the fetus at this SNP position (e.g. when both mother and fetus are heterozygotes for the same alleles), while an informative cell refers to a cell with feto-maternal SNP differences. These terms should be formally defined early on to avoid confusion. However, at line 299, the authors refer to informative SNPs as those where there's a difference between the genotype of the mother and of the putative fetal cell. The term scorable SNP is also used, which here seems to simply refer to a genotyped SNP position that passed quality control.

The following sentence is added in the methods section under variant calling.

Throughout this manuscript, a cell or a SNP is referred to as informative if the putative fetal cell has an allele not carried by the mother (e.g., mother is AA and the putative fetal cell is AB or _B). An uninformative cell does not have alleles not carried by the mother and may be a maternal cell of a fetal cell with inadequate genotyping data.

The legend for Fig. 6 is very consistent with this explanation.

Additional minor suggestions and comments

line 98: "The first step is conventional PCR with a pool of amplicons with bridging adaptors…" should be : "The first step is conventional PCR of a pool of amplicons with bridging adaptors…"

Changed

line 140: "An 8-10 pM diluted denatured…"

changed

line 188: "...three SNPs show a difference between one of the cell…"

changed

line 198: I believe it should be three groups and not four: "The results for cases can be divided into three groups…"

changed

line 214: "...we initially used a more conservative criterion of at least…"

This paragraph is rewritten

line 215: "We found that 68.9 % of genotyped putative fetal cells…"
This paragraph is rewritten

line 228: what does precision of 96.1 % refers to here ? To the authors mean global diagnostic accuracy ? Please mathematically define the precision calculation used.

Changed the text:

“This is equal to a sensitivity [True Positive Rate= TP/(TP+FN) of 91.2% (124/124+12)] and a precision [Positive Predictive Value = TP/(TP+FP) of 96.1% (124/124+5)].”

line 235: "The remaining three cells…"

Changed

line 348: The last sentence ("The fetus does carry the paternal mutation.") seems odd and should be removed or modified.

Deleted sentence.

We think that the revisions above address these weaknesses.

We hope that our revision in its current form is suitable for publication in PLOS ONE.

Sincerely yours,

On behalf of the authors

Xinming Zhuo, Ph.D. and Arthur Beaudet, M.D.
