# Supplementary material for: Use of amplicon-based sequencing for testing fetal identity and monogenic traits with Single Circulating Trophoblast (SCT) as one form of cell-based NIPT
Source: PLoS One. 2021 Apr 15;16(4):e0249695. doi: 10.1371/journal.pone.0249695 (PMC8049273; doi:10.1371/journal.pone.0249695)
Supplement: Attachment — Submitted filename: reply_to_comment.12Mar2021.docx [file pone.0249695.s014.docx]

Dear Editor,

We would like to thank the reviewers for their specific and helpful comments. The manuscript has been improved according to the suggestions from the reviewers.

For specific comments, please see our response below:

Reviewer #3: The authors have addressed most of the issues previously identified, but there remains a few details that need to be clarified.

As requested, the authors have added a sentence defining and clarifying the "informative/uninformative" terminology in the methods section, where this definition is given: "Throughout this manuscript, a cell or a SNP is referred to as informative if the putative fetal cell has an allele not carried by the mother...".
However, at line 249, while refering to Fig. 4, they state that "Cell G106 has only 2 SNP difference in less than 20 informative SNPs...". The axis on Fig. 4 are indeed labelled "number of SNPs not present in mother" and "number of informative SNPs", for the y and x axis respectively.
Therefore, given the definition of informative SNP provided by the authors, I don't understand what can be the difference here between "number of SNPs not present in mother" and "number of informative SNPs". Aren't SNPs not present in mother suppose to be alleles not present in mother ? Please clarify.

We changed the labels in figure 4 and the text accordingly.

“The X-axis indicates the number of variant sites passing coverage cutoff. The Y-axis is the number of informative SNPs in a cell.”

Regarding Fig. 4, there are other inconsistencies between the text and the figure. At line 245, it is said that cells G78, G212, G227 and G232 all have at least four SNPs wich are different from maternal gDNA while on the graph, it seems to be at least five SNPs which are different (of these four cells, G78 and G212 have the lowest number of differences and it seems to be five differences when judging from the axis and the points on the graph). Then it is said that the remaining three cells had 0-2 SNPs which differed from maternal gDNA, while it seems to be rather 0-1 judging from the figure ?
Moreover, the caption for Fig. 4 says: "As expected, the maternal gDNA samples gave no alleles not present in the mother...". This sentence seems odd given that thoughout the paper, we get the impression that maternal gDNA (from WBC) is used as the reference for the mother's genome. How could there be an allele not present in the mother from the data used to establish the mother's genome ? That would be circular ? Was there another reference used ?

We changed typo in the text to “at least five” and “0-1” according to suggest. We also removed the sentence "As expected, the maternal gDNA samples gave no alleles not present in the mother..." to avoid confusion.

Finally, at line 100, the requested change of "...conventional PCR with a pool..." for "...conventional PCR of a pool..." has not been made, even if the authors claim to have made the change in their reply.

We fixed this typo.

We think that the revisions above address these weaknesses.

We hope that our revision in its current form is suitable for publication in PLOS ONE.

Sincerely yours,

On behalf of the authors

Xinming Zhuo, Ph.D. and Arthur Beaudet, M.D.
